# BDNF promotes target innervation of *Xenopus *mandibular trigeminal axons *in vivo*

**DOI:** 10.1186/1471-213X-7-59

**Published:** 2007-05-31

**Authors:** Jeffrey K Huang, Karel Dorey, Shoko Ishibashi, Enrique Amaya

**Affiliations:** 1 Wellcome Trust/Cancer Research UK Gurdon Institute, Tennis Court Road, University of Cambridge, Cambridge, CB2 1QR UK; 2 The Healing Foundation Centre, Faculty of Life Sciences, University of Manchester, Oxford Road, Manchester, M13 9PT, UK

## Abstract

**Background:**

Trigeminal nerves consist of ophthalmic, maxillary, and mandibular branches that project to distinct regions of the facial epidermis. In *Xenopus *embryos, the mandibular branch of the trigeminal nerve extends toward and innervates the cement gland in the anterior facial epithelium. The cement gland has previously been proposed to provide a short-range chemoattractive signal to promote target innervation by mandibular trigeminal axons. Brain derived neurotrophic factor, BDNF is known to stimulate axon outgrowth and branching. The goal of this study is to determine whether BDNF functions as the proposed target recognition signal in the *Xenopus *cement gland.

**Results:**

We found that the cement gland is enriched in BDNF mRNA transcripts compared to the other neurotrophins NT3 and NT4 during mandibular trigeminal nerve innervation. BDNF knockdown in *Xenopus *embryos or specifically in cement glands resulted in the failure of mandibular trigeminal axons to arborise or grow into the cement gland. BDNF expressed ectodermal grafts, when positioned in place of the cement gland, promoted local trigeminal axon arborisation *in vivo*.

**Conclusion:**

BDNF is necessary locally to promote end stage target innervation of trigeminal axons *in vivo*, suggesting that BDNF functions as a short-range signal that stimulates mandibular trigeminal axon arborisation and growth into the cement gland.

## Background

Peripheral axon targeting comprises at least 2 morphologically distinct growth states: directional elongation, followed by terminal arborisation at the targets [1]. Target derived diffusible factors are known to control the outgrowth and branching of growing axons [2]. In *Xenopus*, the mandibular trigeminal nerve extends as fasciculated neurites for a visibly long distance toward the anterior facial epithelium, where in the vicinity of the cement gland, trigeminal axons turn ventrally, arborise and grow into the ventral posterior domain of the cement gland [3]. The cement gland is a transient embryonic tissue, made up of highly pigmented, mucous secreting cells. These cells transmit mechanosensory information via pressure sensitive receptors in trigeminal axon terminals to activate tonic inhibition response in the swimming tadpole [4,5]. Honore and Brivanlou have previously demonstrated that the surgical deletion of cement glands from *Xenopus *embryos resulted in mandibular trigeminal nerve targeting error, and proposed that a cement gland-derived chemoattractive signal operates from a short distance to control the branching and growth of mandibular trigeminal axons to the cement gland [6].

It is well known in co-culture experiments that directed outgrowth of trigeminal axons could be stimulated by tissue derived chemoattractants, termed Maxillary Factor from target maxillary/mandibular tissues [7]. It has since been demonstrated that Maxillary Factor comprises the neurotrophins BDNF and NT3 [8]. Neurotrophins are a family of secreted ligands, including NGF, BDNF, NT3 and NT4 that bind to designate Trk receptors in the nervous system to promote neuronal survival [9,10]. Previous studies showed that mice deficient in BDNF or NT3 displayed a profound loss of sensory neuron populations in the trigeminal ganglia and spinal cord, and die shortly after birth [9-12]. Furthermore, these mice did not exhibit any defect in the projection of trigeminal axons [8]. However, it remained unclear whether target-derived neurotrophins might function locally to promote the end stage targeting of trigeminal axons *in vivo*. In *in vitro *growth cone turning assays, growth cones from isolated spinal cord neurons could orient toward a directional gradient of neurotrophins emanating from a micropipette [13]. Moreover, regional overexpression of neurotrophins in the *Xenopus *central nervous system could promote localised growth and branching from neurotrophin responsive axons [14]. Additionally, neurotrophins when applied ectopically in mammalian slice cultures can promote sensory axon outgrowth from the spinal cord into the periphery [15]. Since the cement gland is a well-defined target for mandibular trigeminal axons, *Xenopus *might provide a useful model for the analysis of neurotrophin function in axon-target interaction *in vivo*. Recently it has been demonstrated that TrkB, the receptor for BDNF and NT3 is highly expressed by trigeminal and spinal sensory neurons in *Xenopus *embryos, which suggests that neurotrophins might play a role in peripheral sensory axon development [16]. Here we determine whether the *Xenopus *cement gland expresses neurotrophins, and focus on the role of BDNF during mandibular trigeminal axon target innervation *in vivo*.

## Results

### Expression of BDNF in the *Xenopus *cement gland during mandibular trigeminal axon development

**Figure 1:**
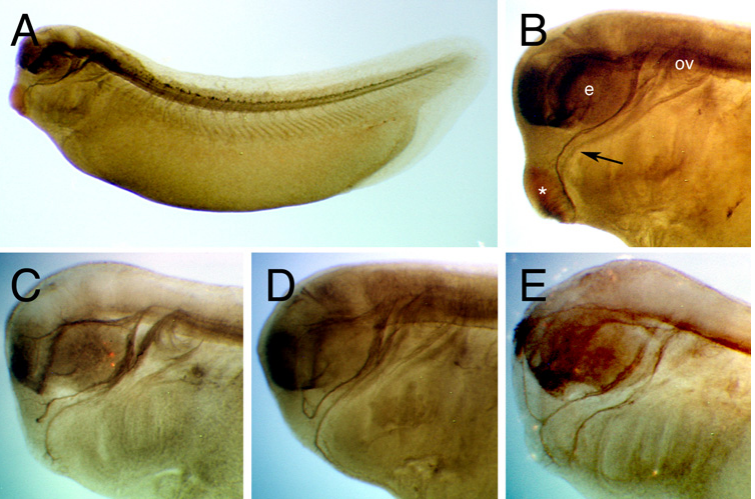
***Xenopus *mandibular trigeminal nerve innervates the cement gland**. (A) Whole mount immunostaining of a *Xenopus *embryo (St. 29) labeled with β-tubulin antibody and cleared in Murray's clearing solution reveals the embryonic axonal network, including the prominent mandibular trigeminal nerve. (B) The mandibular trigeminal nerve (arrow) extends between the eye vesicle (e) and otic vesicle (ov), and then turns ventrally before terminating at the ventral region of the cement gland (asterisk). Deletion of cement glands results in the loss of trigeminal targeting, where the axons either (C) stop growth, or extended (D) dorsally or (E) ventrally. Because the embryos are transparent, trigeminal axons on the opposite side could also be detected. In the cement gland null embryos, trigeminal axons on either side of the face do not necessarily project erroneously in the same directions.

The mandibular trigeminal nerve is a prominent cranial nerve that innervates the *Xenopus *cement gland (Figure 1A). During development, it exits and projects from the trigeminal ganglion as fasciculated neurites towards the cement gland. Once near the cement gland, the axons extend ventrally and then enter the ventral domain of the cement gland as branched fibers [6] (Figure 1B). To confirm the presence of local axon targeting signals in the cement gland, we repeated Honore and Brivanlou's experiment of surgically deleting the cement gland in developing embryos at St. 18, and analysed mandibular trigeminal nerve morphology at St. 29, the stage of axon target innervation by whole mount immunostaining. Consistent with their observation [6], we detected the disruption of mandibular trigeminal nerve targeting in embryos lacking the cement gland (n = 10). Without intact cement glands, mandibular trigeminal nerves extended normally to the anterior facial epithelium, but then either stopped before the site of cement gland deletion (30%; Figure 1C), grew away to the dorsal facial epithelium (20%) or to the ventral epithelium (50%; Figure 1D and 1E). Furthermore, most of the axons remained fasciculated and axon terminals did not arborise. This observation suggests that the cement gland is likely to provide a short range signal to promote the end stage targeting of mandibular trigeminal axons by stimulating branching and growth into the ventral domain of the cement gland.

**Figure 2:**
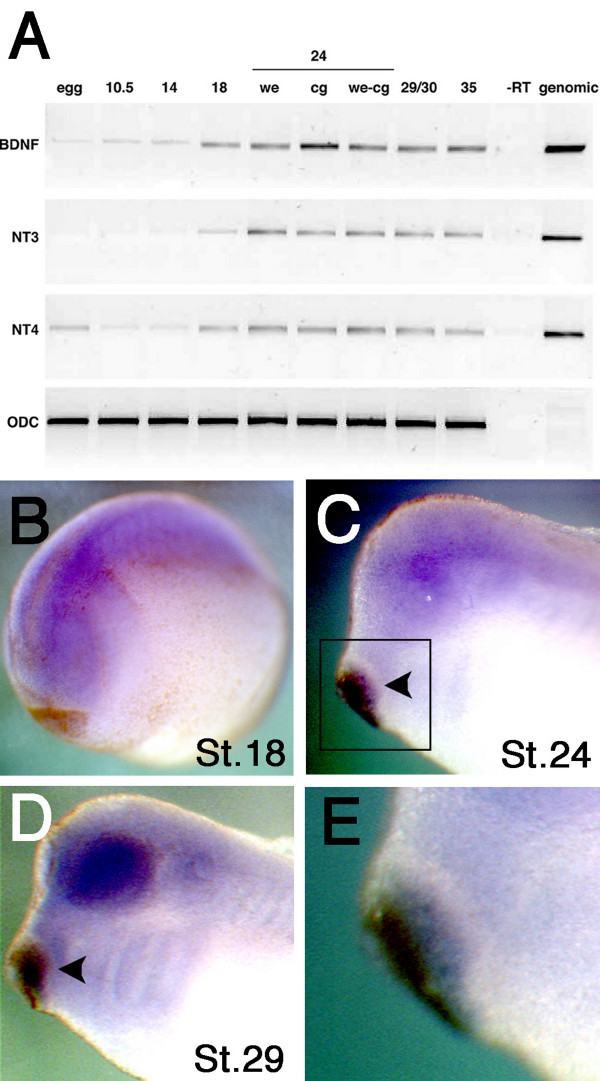
**Detection of BDNF transcripts in the *Xenopus *cement gland**. (A) RT-PCR shows that BDNF, NT3 and NT4 were detected throughout all stages of embryonic development. Expression of these genes increases during neurulation (St. 18). The BDNF transcript was particularly enriched in the cement gland compared to the other neurotrophin transcripts at tailbud stage (St. 24). The amount of input cDNA was confirmed with primers generated against the housekeeping gene, ornithine decarboxylase (ODC) to ensure equal loading. (B) Whole mount *in situ *hybridisation shows that BDNF mRNA are initially detected in the neural plate at St. 18. (C) At St. 24, BDNF is detected at the posterior region of the cement gland (arrowhead), which corresponds to the time of trigeminal nerve innervation. (D) At St. 29, BDNF expression remains in the cement gland and can also be detected in the eye and otic vesicles. (E) Inset from (C) shows the detection of BDNF mRNA in the cement gland at St. 24. we = whole embryo, cg = cement gland, we-cg = whole embryo without cement gland.

Given that neurotrophins have been suggested to mediate mammalian peripheral trigeminal axon outgrowth *in vitro *[8,17], we asked when and where neurotrophins are expressed in *Xenopus *embryos. We found that BDNF, NT3 and NT4 are expressed in early developing embryos (Figure 2A). Additionally, their expression increased at St. 18 during the period of primary neuronal development, and appeared to remain constant throughout all of the subsequent developmental stages examined. During development, the *Xenopus *mandibular trigeminal axons grow out toward the cement gland at St. 24 and terminate at the cement gland by St. 29 [6]. When we examined the expression of neurotrophins in cement glands of St. 24 embryos, we found that the cement gland is enriched in BDNF mRNA, compared to NT3 and NT4 (Figure 2A). By whole mount *in situ *hybridisation we detected BDNF mRNA at the neural plate in neurula stage embryos (St. 18) (Figure 2B), and in the posterior region of the cement gland in tailbud stage embryos at St. 24 (Figure 2C and 2E) and St. 29 (Figure 2D). The detection of BDNF mRNA in the cement gland suggests that BDNF might mediate mandibular trigeminal axon target innervation in *Xenopus*.

### Phenotype of *Xenopus *embryos after BDNF knockdown

**Figure 3:**
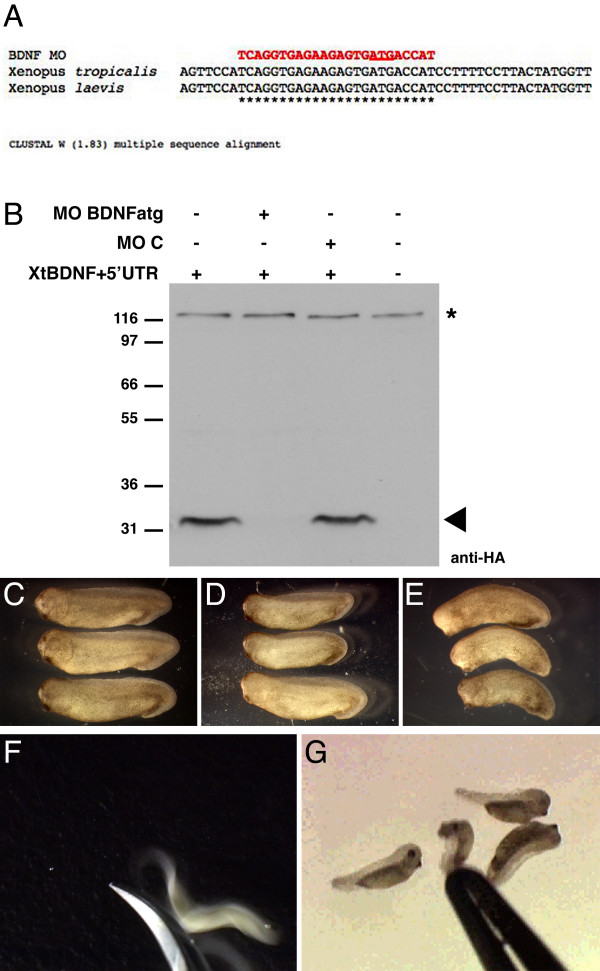
**Effect of BDNF antisense morpholino oligonucleotides (MO) on *Xenopus *morphology and behaviour**. (A) BDNF MO was designed against the 5' UTR and start ATG region (underlined). This region was 100% conserved between *X. laevis *and *X. tropicalis*. Asterisks indicate conserved bases. (B) Embryos were injected with 250 pg of XtBDNF-HA mRNA alone or with 20 ng of MO BDNFatg or the control MO (MOC). Embryos were harvested at St 12 and proteins extracts analysed by Western blotting. Xt BDNF was detected using anti-HA antibodies (arrowhead). The asterisk indicates a non-specific band from the same blot, which ensures equal loading in each lane. (C) Uninjected *X. laevis *embryos at St. 29. (D) Control morpholino (MOC) injected embryos at St. 29 developed normally. (E) BDNF morphants appeared slightly truncated at St. 29. (F) An uninjected tadpole with normal mechanosensory response, which escaped when probed with forceps. (G) BDNF morphants exhibited impaired mechanosensory response and did not escape when probed.

To examine BDNF function during *Xenopus *nerve development, we used an antisense morpholino oligonucleotide (MO) approach to knockdown BDNF expression. We identified the BDNF gene in the genome in the JGI *Xenopus tropicalis *genomic database [18]. We then obtained the genomic sequence of BDNF and designed a MO (MO BDNFatg) against a 24 nucleotide sequence in the 5' region of XtBDNF mRNA, including the start codon (Fig 3A). This region is 100% conserved between *Xenopus laevis *and *Xenopus tropicalis *BDNF. Furthermore, it is highly conserved in all of the BDNF genes examined in other vertebrates and mammals

First, we tested whether MO BDNFatg inhibited translation of an HA-tagged XtBDNF construct in *X. laevis *embryos. The HA tag was added to the C-terminus of XtBDNF (BDNF-HA), and the region surrounding the start of translation was not altered. When the BDNF-HA RNA was injected alone in *X. laevis *embryos, a specific band at 34 kDa was detected by Western blot analysis (Figure 3B, arrowhead). However, the expression of BDNF-HA was completely inhibited by co-injection of MO BDNFatg. As control, we used an oligonucleotide designed against the *X. tropicalis *Sprouty2 gene, containing 4 mismatch nucleotides (MOC), which has previously been shown not to affect *Xenopus *development [19]. We found that MOC had no effect on the translational efficiency of the HA-tagged BDNF construct (Figure 3B). This showed that the BDNF MO was highly effective in knocking down BDNF translation.

To examine the effects of BDNF knockdown on axon development, we injected MOs into *X. laevis*, and *X. tropicalis *embryos. We found that all of the MO BDNFatg injected embryos (n = 68 *X. tropicalis*, n = 30 *X. laevis*) developed without visible morphological abnormalities up to the early tailbud stage (St. 24). These animals were similar in appearance to uninjected embryos (n = 56 *X. tropicalis*, n = 50 *X. laevis*) and MOC embryos (n = 22 *X. tropicalis*, n = 15 *X. laevis*). At St. 28/29, both *X. laevis *and *X. tropicalis *injected with MO BDNFatg appeared slightly truncated in appearance (Figure 3E) compared to uninjected (Figure 3C) and control MOC injected embryos (Figure 3D). At St. 29, *Xenopus *embryos normally twitch and escape if lightly touched with an object (Figure 3F). Compared to uninjected and control MOC injected embryos which exhibit normal mechanosensory reflex (Additional file 1 and 2), we observed that the BDNF morphants appeared paralysed (Figure 3G and Additional file 3). Despite the lack of visible sensory reflex, the morphants continued to develop until the hatching stage (St. 32). These results show that BDNF knockdown results in consistent phenotype and behaviour in both *X. tropicalis *and *X. laevis *embryos.

### Analysis of mandibular trigeminal axon targeting after BDNF knockdown

**Figure 4:**
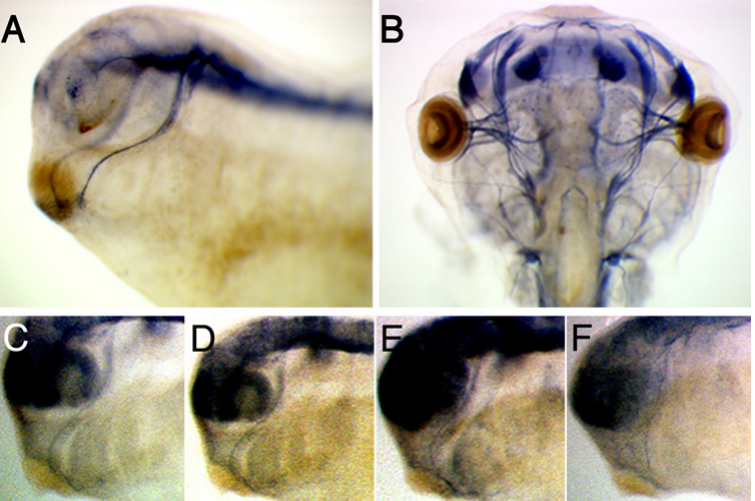
**MO BDNFatg injected into N-PLAP transgenic *X. tropicalis *embryos exhibit possible disruption in mandibular trigeminal axon targeting**. (A) A transgenic *X. laevis *embryo (St. 29) expressing N-PLAP following alkaline phosphatase reaction demonstrates the neurospecific labeling of CNS axons. The mandibular trigeminal nerve can be observed to project to the cement gland where terminal arborisation occurs. (B) The detection of cranial nerves in a transgenic tadpole (St. 47). (C) An uninjected *X. tropicalis *transgenic embryo (WT) displaying normal trigeminal nerve projection. (D) A MOC injected transgenic embryo and (E) a MO NT3 injected embryo show normal trigeminal nerve projection. (F) A MO BDNFatg injected transgenic embryo shows that the trigeminal axons appeared thinner and possibly less fasciculated. Some of the axons did not extend to the posterior cement gland where they normally innervate.

We next determined if mandibular trigeminal axon outgrowth was disrupted in MO BDNFatg injected embryos at St. 29. The *X. tropicalis *embryos used in our experiment were obtained from sibling mating of a recently generated transgenic line which expresses human placental alkaline phosphatase (PLAP) under the control of the neuronal specific β-tubulin promoter. The advantage in using this line is that PLAP is a heat stable and histochemically detectable enzyme [20], which enables the rapid detection of axonal projections *in situ *in whole embryos by a simple alkaline phosphatase (AP) reaction at any stage of development (Figure 4A and 4B). In uninjected embryos (n = 23), normal projection of mandibular trigeminal axons was observed, where fasciculated axons extended to the target tissue and then turned ventrally toward the ventral domain of the cement gland (Figure 4C). In MOC embryos (n = 16), as well as in MO NT3 injected embryos (n = 25), projection of mandibular trigeminal axons also appeared normal (Figure 4D and 4E). In MO BDNFatg injected embryos (77.8%, n = 18), the majority of mandibular trigeminal axon appeared to project straight to the cement gland area, and did not turn toward the ventral domain of the cement gland, where they normally innervate (Figure 4F). Additionally, premature axon defasciculation was occasionally observed among the morphants (28.6%).

**Figure 5:**
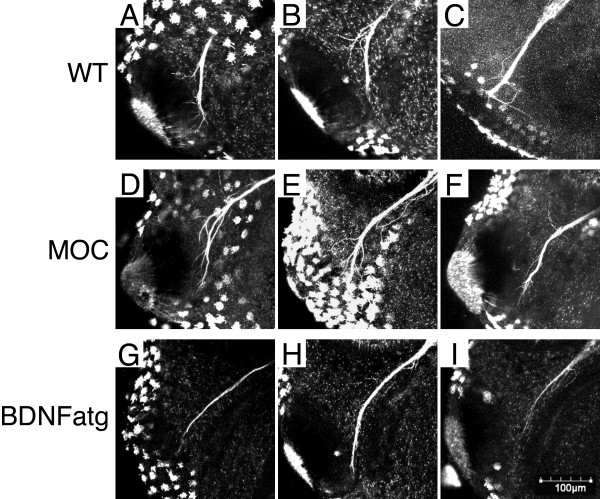
**Decreased axon terminal arborisation in cement glands of BDNF morphants**. Whole mount immunofluorescent staining of St. 29 *X. laevis *embryos with anti-acetylated α-tubulin shows trigeminal axon arborisation and growth into the cement gland in (A), (B) and (C) uninjected, and (D), (E) and (F) MOC injected embryos. (G), (H) and (I) In MO BDNFatg injected embryos trigeminal axons extended to the cement gland but did not arborise or grow into the cement gland.

It was however difficult to assess the morphology of trigeminal axon terminals at the cement gland by AP reaction. To examine in detail whether trigeminal axons terminated into cement glands of BDNF morphants, we performed whole mount immunostaining by using antibody against acetylated α-tubulin on MO injected embryos. At St. 29 the embryos were analysed by scanning confocal microscopy. In uninjected (n = 14; Figure 5A, B and 5C) and MOC injected embryos, we observed that the trigeminal axon terminals arborised and entered the ventral cement gland (n = 13; Figure 5D, E and 5F; Table 1). However, in MO BDNFatg injected embryos (n = 25), we observed that most trigeminal axon terminals remained fasciculated and did not arborise or grow into the cement gland (Figure 5G, H and 5I). Axonal arborisation was only detected in 16% of the morphants. This observation demonstrates that BDNF expression is necessary for trigeminal axon target innervation at the cement gland. Furthermore, it suggests that terminal arborisation might be necessary for trigeminal axon growth into the cement gland.

**Table 1:** Analysis of mandibular trigeminal axon arborisation after *in vivo *cement gland swaps.

	**n =**	**No Arborisation**	**Arborisation**
**WT**	14	0%	100%
**MOC**	13	15%	85%
**MO BDNFatg**	25	84%	16%
**MOC CG/WT Emb**	7	28%	72%
**WT CG/MOC Emb**	6	17%	83%
**MO BDNFatg CG/WT Emb**	23	87%	13%
**WT CG/MO BDNFatg Emb**	14	21%	79%

CG = cement gland, Emb = embryo, WT = uninjected

**Figure 6:**
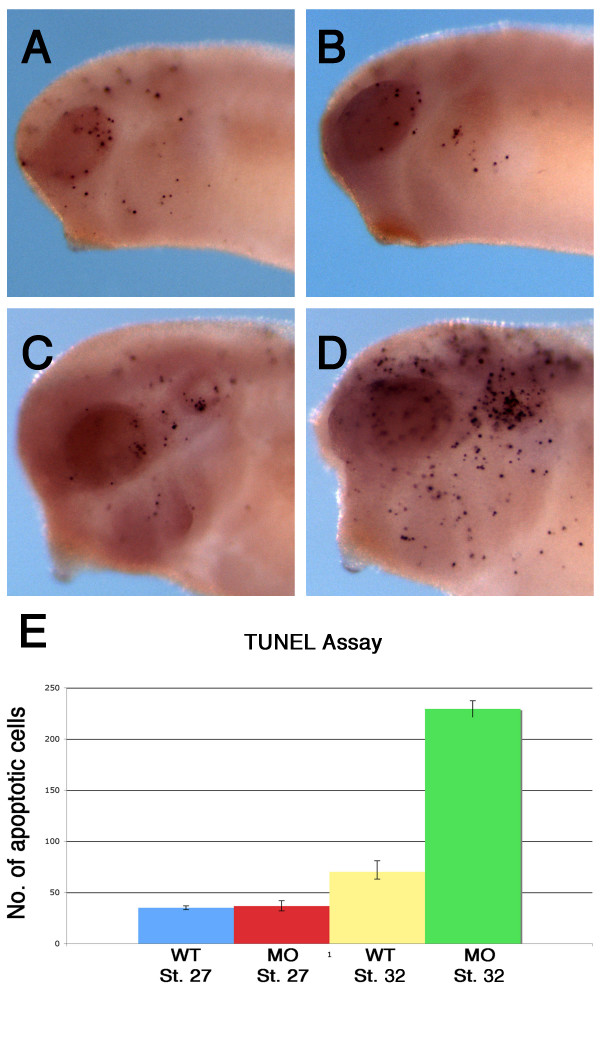
**Increased apoptosis observed in the head region of BDNF morphants**. Whole mount TUNEL staining of uninjected embryos at (A) St. 27 and (B) 32. In MO BDNFatg injected morphants, cell death activity in the head region was comparable to those in uninjected embryos at (C) St. 27, but number of cells undergoing apoptosis increased significantly at (D) St. 32. (E) Quantitation of cells undergoing apoptosis shows a 3-fold increase of cell death activity in St. 32 BDNF morphants.

However, because BDNF is also crucial for the survival of sensory neuron populations [21], it remained possible that the disruption of mandibular trigeminal axon target innervation in the morphants was due to an increase in overall cell death throughout the morphants rather than directly from the loss of BDNF expression in the cement gland. To address this issue, we performed a whole mount TUNEL staining to assess cell death activity in BDNF morphants. We found that the number of cells undergoing cell death in the head region of BDNF morphants (n = 10) was comparable to those observed in uninjected embryos (n = 10) during the period of mandibular trigeminal axon targeting into the cement gland (St. 27) (Figure 6A, B and 6E). At hatching stage (St. 32), we detected at least a 3 fold increase in cell death activity of BDNF morphants (n = 10) compared to uninjected embryos (n = 10), which is 12 hours after mandibular trigeminal axons should have terminated into the cement gland (Figure 6C, D and 6E). The disruption of mandibular trigeminal axon targeting in BDNF morphants was therefore likely due to the loss of BDNF signalling and independent from cell death activities.

### Local BDNF expression promotes mandibular trigeminal axon arborisation *in vivo*

**Figure 7:**
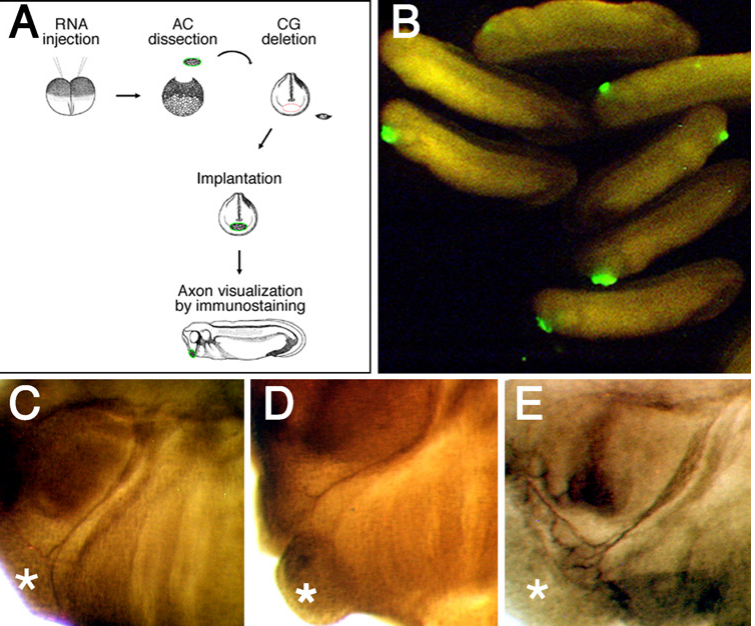
**BDNF expressed ectodermal animal cap grafts stimulate mandibular trigeminal axon arborisation**. (A) Diagram of the *in vivo *cement gland substitution assay. (B) Successfully grafted embryos were identified by GFP expression. (C) Neither GFP^+ ^nor (D) NT3^+ ^grafts stimulated trigeminal axon arborisation or growth onto the grafts. (E) BDNF^+ ^graft stimulated axon terminal arborisation onto the graft. AC = animal cap, CG = cement gland, Asterisk = graft.

We next determined whether mandibular trigeminal axon target innervation could be promoted by local BDNF expression by using an "*in vivo *cement gland substitution assay" (Figure 7A) [6]. Experimental deletion of the cement gland causes the failure of mandibular trigeminal nerve targeting (Figure 1C, D and 1E). A previous study showed that target innervation can be "rescued" by substituting the cement gland with an ectodermal animal cap explant expressing follistatin [6]. We asked if ectodermal animal cap grafts expressing BDNF when positioned in place of cement glands, could prevent trigeminal axon targeting error by stimulating local axon arborisation and growth into the grafts. *In vitro *transcribed mRNAs expressing either green fluorescent protein (GFP), or BDNF and GFP, were first injected into both blastomeres of *Xenopus *embryos at the 2-cell stage. At blastula stage (St. 8), ectodermal animal cap explants were excised and grafted onto a set of uninjected embryos at the late neurula stage (St. 18/19), which had their cement glands removed. At this stage, trigeminal ganglia have not projected their axons. The grafted embryos were then allowed to heal and develop until the late tailbud stage (St. 29). Embryos that contained intact grafts and GFP reporter expression (Figure 7B) were further analysed for trigeminal axon targeting by whole mount immunostaining with an antibody against β-tubulin. We found that the GFP expressed grafts did not prevent trigeminal axon targeting error in *Xenopus *embryos, as the axons either stopped short of the target (22%), extended dorsally toward the eye (33%), or ventrally toward the heart (45%) (n = 9; Figure 7C and Additional file 4). Furthermore, none of the grafts promoted visible axon terminal arborisation or growth into the graft. This observation was similar to that observed when cement glands were experimentally deleted (Figure 1C, D and 1E and [6]). Moreover, this result also demonstrates that secreted factors that might normally be expressed by animal cap cells do not influence or have a direct effect on trigeminal axon targeting. We also tested the possibility for NT3 expressed grafts to promote trigeminal axon targeting (n = 11) by performing the same assay, but found that NT3 was unable to significantly stimulate axon arborisation or growth into the graft (Figure 7D and Additional file 4). However, when we examined embryos containing BDNF expressed grafts, we found that the grafts promoted axon arborisation at the grafts in 82% of the embryos (n = 17; Figure 7E and Additional file 4). This suggests that peripheral expression of BDNF could stimulate axon arborisation towards the site of its expression. Furthermore, this observation demonstrates that BDNF acts locally on trigeminal axons because the axons remained fasciculated during their projection, and target induced branching was only observed at the grafts.

**Figure 8:**
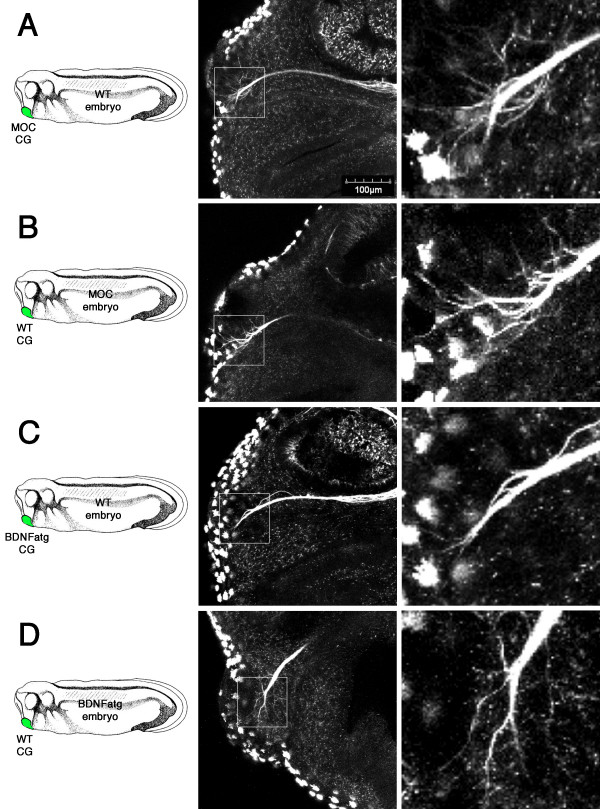
**Wildtype cement gland grafts rescued mandibular trigeminal axon target innervation in MO BDNFatg injected embryos**. *In vivo *cement gland swap experiments were performed between MOC injected embryos and uninjected embryos (WT). (A) MOC cement gland placed onto an uninjected embryo exhibits trigeminal axon arborisation and growth into the cement gland. (B) Similar observation was made when wildtype cement gland was placed onto a MOC injected embryo. *In vivo *cement gland swaps were also performed between BDNF morphants and uninjected embryos. (C) MO BDNFatg cement gland placed onto an uninjected embryo shows trigeminal arborisation at the cement gland was barely detectable. (D) Wildtype cement gland placed onto a MO BDNFatg injected embryo shows that trigeminal axons were able to arborise and enter the cement gland.

While the previous experiment showed that BDNF is sufficient to stimulate terminal arborisation, we wanted to know whether BDNF expression within the cement gland *in vivo *is necessary for target recognition by trigeminal axon terminals. To address this, we performed an "*in vivo *cement gland swap" experiment by swapping cement glands between uninjected embryos and morpholino injected embryos at St.18/19 and then analysed mandibular trigeminal axon targeting at St. 29 by scanning confocal microscopy. First, we examined cement gland swaps between uninjected and MOC injected embryos. We found that MOC injected cement glands grafted onto wildtype embryos did not perturb mandibular trigeminal axon targeting, as the axons were able to arborise and grow into the cement gland grafts (72% arborisation, n = 7; Figure 8A and Table 1). A similar observation was also made when wildtype cement glands were grafted onto MOC injected embryos (83% arborisation, n = 6; Figure 8B and Table 1). We next examined cement gland swaps between uninjected and MO BDNFatg injected embryos. We found that BDNF MO injected cement glands, when grafted onto wildtype embryos, did not promote significant axon arborisation or growth into the cement glands (13% arborisation, n = 23; Figure 8C and Table 1). This observation was similar to that observed in MO BDNFatg injected embryos, and further demonstrates that the failure of axon arborisation at the cement gland in BDNF morphants was specific to BDNF knockdown in the cement gland and not due to a general effect elsewhere in the embryo. Furthermore, we found that wildtype cement glands, when grafted onto MO BDNFatg injected embryos, were able to rescue trigeminal axon target innervation from BDNF deficient embryos by promoting axon arborisation and growth into the grafted cement gland (79% arborisation, n = 14; Figure 8D and Table 1). This demonstrates that trigeminal axon terminals in BDNF morphants remained responsive to target recognition signals from the cement gland. Furthermore, this suggests that BDNF expression at the cement gland is necessary for mandibular trigeminal axon target innervation.

## Discussion

### *Xenopus *mandibular trigeminal nerve as a model for *in vivo *axon-target interaction studies

Previous *in vivo *studies in mice determined BDNF as a trophic factor to promote sensory axon development and survival. But because BDNF null mice die soon after birth, it has not been possible to show *in vivo *whether BDNF functioned as a short range signal to stimulate axon target innervation [8-10]. There are several advantages in using *Xenopus *to study the roles of BDNF on axon targeting. The *Xenopus *mandibular trigeminal nerve and its target, the cement gland, provide a simple and useful *in vivo *system to examine the molecular basis of axon-target interaction in vertebrates. *Xenopus *is an excellent model for embryological studies due to the advantage of its rapid external development, and ability for micromanipulations, grafting and explant cultures. Additionally, it is amenable to genetic manipulations by germ-line transgenesis [22], as well as effective antisense morpholino oligonucleotide analysis [23,24]. By using these techniques, we showed that BDNF expression in the *Xenopus *cement gland functioned to locally promote end stage target innervation of mandibular trigeminal axons *in vivo*. Furthermore, because the developmental staging in *Xenopus *embryos is well characterised, we were able to precisely separate the effect of BDNF on target innervation from its effect on neuronal survival by analyzing distinct developmental time points.

### BDNF is a short-range signal to locally stimulate mandibular trigeminal axon arborisation and target innervation

We found that BDNF knockdown did not affect the overall projection of mandibular trigeminal axons, but prevented their arborised growth into the target cement gland. Our data suggest that BDNF expressed at and near the cement gland functions to promote changes in axonal morphology necessary for trigeminal axon arborisation and growth into the cement gland. In the absence of BDNF, trigeminal neurons fail to arborise and enter the cement gland. By performing cement gland swapping experiments between control and experimental knockdown embryos, we were able to show that BDNF expression is required locally within the cement gland for terminal arborisation of the trigeminal nerves into the cement gland. Finally, transplantation of tissue overexpressing BDNF near the trigeminal nerve results in excessive branching and growth of the nerves into the BDNF expressing transplant, suggesting that BDNF is an important component of the local trigeminal nerve targeting signal, proposed by Honore and Brivanlou [6]. This finding is consistent with the findings of Nosrat and colleagues [25] and Albers and colleagues [26], who have shown that ectopic overexpression of BDNF in mice can lead to aberrant branching and sprouting of axons at the mammalian tongue.

In *Xenopus *embryos, trigeminal and spinal sensory neurons express TrkB during development [16]. BDNF is known to bind to the receptor tyrosine kinase TrkB, which activates several downstream signalling cascades, including the Ras-Mek-Erk and the PI3K/AKT pathways [27,28]. Activation of TrkB signalling might trigger local changes in actin cytoskeleton polymerisation within growth cones to promote arborisation of trigeminal axons [29]. BDNF can also bind to the low-affinity neurotrophin receptor p75NTR [30,31]. p75NTR plays a very important role in sensory neuron survival, outgrowth and innervation [32,33], and can function in conjunction with or independently of Trk receptors [31,34]. It is thought that the specificity of TrkB activation by BDNF increases in the presence of p75NTR [30]. It is possible that at the cement gland, BDNF binds to both TrkB and p75NTR on the growth cone to promote mandibular trigeminal axon innervation and survival.

### Possible involvement of other neurotrophins during mandibular trigeminal axon outgrowth

We detected the expression of other neurotrophins NT3 and NT4 in the cement gland, although their expression level was not enriched there, compared to BDNF. In mammalian *in vitro *axon outgrowth assays, NT3 was suggested as the dominant neurotrophin that stimulates axon outgrowth [8]. It remains possible that NT3 and NT4 might also play a role in mandibular trigeminal axon development in *Xenopus*. However we tested the effect of NT3 on mandibular trigeminal axon targeting by using the *in vivo *cement gland graft experiment, and found that NT3 did not appear to stimulate visible arborisation or target innervation. We also tested NT3 MO in *Xenopus*, and found that it did not have a major effect on trigeminal axon targeting at the stages we examined (St.29), and the embryos were not paralysed (See Additional file 5). In contrast, mandibular trigeminal axons rarely arborised or entered cement glands where BDNF expression was knocked down, therefore BDNF appears to be the dominant local signal that promotes local axon arborisation at the cement gland. We did, however, observe occasional arborisation at the cement gland in some BDNF morphants (16%). It is possible that the occasional arborisation was caused by other signals, or by low level BDNF expression from incomplete BDNF knockdown at the cement gland. The nerve growth factor, NGF, is known to stimulate axon outgrowth from avian and mammalian trigeminal neurons in culture [35,36]. However, NGF is not likely to stimulate mandibular trigeminal axon innervation in *Xenopus *embryos, as neurons isolated during the stages when trigeminal nerves innervate the cement gland are not responsive to NGF [13,37], and do not express the NGF receptor TrkA [38]. Nevertheless we cannot exclude a role for NGF in later stage maintenance of trigeminal neurons, as NGF is known to be expressed in the nervous system in later stage tadpoles [39]. Another neurotrophin family member, glial derived neurotrophic factor, GDNF has also been suggested to play a role in the outgrowth and maintenance of peripheral trigeminal axons [40,41]. Recently, GDNF was isolated in *Xenopus *and shown to be expressed in the maxillary facial region of *Xenopus *embryos during development [42]. But it does not appear to be expressed in the cement gland. It is possible that GDNF does not regulate target innervation, but plays a role in the guidance or maintenance of mandibular trigeminal axons.

### BDNF mediated target innervation might be necessary for mechanosensory function

We also found that all of the BDNF morphants appeared paralysed. This might be due to the loss of BDNF responsive neurons within the trigeminal ganglia and spinal cord, since BDNF expression is necessary for sensory neuron survival and maintenance [9,10]. Neurotrophins and their receptors have been suggested to play a role in the development of cutaneous mechanoreceptors [43]. Recently, BDNF expression in mouse was shown to regulate the development of slowly adapting mechanoreceptors in cutaneous sensory neurons, independently from its role in promoting cell survival [44]. It is possible that local BDNF expression might provide a signal to stimulate mechanoreceptor differentiation in *Xenopus *sensory axons, but this remains to be determined.

## Conclusion

BDNF is expressed in the cement gland during mandibular trigeminal axon development, and is required to directly stimulate the end stage targeting of *Xenopus *mandibular trigeminal axons *in vivo*. The disruption of BDNF signalling resulted in mandibular trigeminal axon targeting failure by preventing their arborisation and growth into the cement gland. BDNF's role in end stage axon targeting appeared to be independent from its role in cell survival.

## Methods

### RT-PCR

Total RNA was extracted from embryos using TRIzol according to manufacturer's instructions (Invitrogen). Ten cement glands gave similar total RNA concentration as one whole embryo. After reverse transcription using Superscript II (Invitrogen), cDNAs were subjected to PCR using the following primers in 5' to 3' direction: *BDNF forward*: CAA GTA CCT TTG GAG CCA CC, *BDNF reverse*: CTA TCC ATG GTG AAA GCC CGC ACG; *NT3 forward*: ATG TGT CTA TTC TTA TCC ACC GCC, *NT3 reverse*: CAT CAA AGC ACC ATA CCA AAG CC; *NT4 forward*: CAT TGC TTT TTG TCT ACA CCT CGG, *NT4 reverse*: TCA TAC TGT TGT GCC ATC TGC; *ODC *[45]. Samples were taken after 29 cycles of amplification for BDNF, NT3 and NT4 in the linear range of amplification.

### Whole-mount *in situ *hybridisation and immunohistochemistry

*In situ *hybridisation was performed following the protocol of Harland [46]. Antisense probes were generated from pX112-13 containing partial *Xenopus *BDNF cDNA (S. Cohen-Cory, UC Irvine). Immunostaining was performed as previous described [47]. Briefly, embryos were fixed in 4% paraformaldehyde and stored in methanol at -20°C until use. The embryos were then rehydrated and washed twice with BBT (1%BSA, 0.1%Tx-100 in 1 × PBS) for 1 hour, once with BBT+5% heat treated lamb serum for 1 hour, and then incubated overnight with mouse monoclonal antibodies against β-tubulin isotype I+II (Product No. T8535, Sigma) for peroxidase detection, or anti-acetylated α-tubulin (Product No. T-6793, Sigma) for immunoflourescence detection. The embryos were then washed four times with BBT for 1 hour, once with BBT+5% heat treated lamb serum for 1 hour, and then incubated overnight with secondary anti-mouse antibodies conjugated to HRP (Molecular Probes) or to Alexa Fluor 568 (Molecular Probes). Embryos then were washed once with BBT for 1 hour and 3 times with PBT (0.1%Tween in 1 × PBS) for 1 hour, then with PBT overnight. Then the embryos were transferred to methanol before peroxidase detection by ImmnunoHisto Peroxidase Detection kit (Pierce), or scanning confocal microscopy analysis. For viewing, the embryos were cleared in Murray's clearing solution (2:1, Benzyl Benzoate:Benzyl alcohol).

### Morpholino oligonucleotides

Antisense morpholino oligonucleotides (MOs) were purchased from Gene Tools LLC. Genomic sequence encompassing the start ATG region of *X. tropicalis *BDNF was obtained from Ensembl [48]: 5'-ATGGTCATCACTCTTCTCACCTGA-3'. As a control, a morpholino designed against the ATG of Xt Sprouty 2 containing 4 mismatch was used: 5'-GCCTTTTAGTACTCTCGTGTCCTTC-3' [19]. *X. tropicalis *embryos were injected with 5–8 ng total MOs along with Micro-ruby fluorescent dextran (Invitrogen) at the one cell stage. *X. laevis *embryos were injected with 20 ng total MOs together with *in vitro *transcribed GFP mRNA into both blastomeres at the 2 cell stage. Embryos that were positive for fluorescence were further analysed for trigeminal axon targeting by whole mount immunostaining.

### Western blot

pCS2 5'UTR+XtBDNF was generated by PCR on genomic DNA using the following primers 5' CTG GAT CCA GAT GTT CCT AAT TCC TGT 3' and 5' GAC CAT TAA AAG GGG AAG ATA TCC ATA CGA TGT TCC AGA TTA CGC TTG AGA ATT CAG 3'. After digestion with EcoRI and BamHI, the PCR product was subcloned into pCS2 and verified by sequencing. Synthetic mRNA (250 pg) derived from this plasmid was injected in 1-cell stage *X. laevis *embryos alone or with 20 ng of MO BDNFatg or MOC. Embryos were harvested at St 12 and homogenized in Lysate Buffer (150 mM NaCl, 20 mM Tris pH7.5, 5 mM EDTA/EGTA and Proteases Inhibitors (Roche)). The equivalent of one embryo was loaded and fractionated on an SDS-PAGE gel. After electrophoresis, proteins were transferred to PDVF membrane (Millipore) and membranes were probed with anti-HA HRP conjugated (clone 3F10, 1667475, Roche) followed by ECL (Amersham).

### Whole mount TUNEL staining

Fixed embryos were washed twice with PBTw (0.2% in 1 × PBS) and twice with 1 × PBS. For end labelling, the embryos were washed with terminal deoxynucleotidyl transferase (TdT) buffer (GibcoBRL) for 30 minutes and then incubated with 0.5 μM digoxigenin-UTP and 150 U/ml TdT in TdT buffer overnight. The embryos were then washed twice with 1 × PBS containing 1 mM EDTA for 1 hour at 65°C, then four times with 1 × PBS for 1 hour at room temperature. For detection and chromogenic reaction, embryos were washed with PBT, blocked with PBT+20% goat serum, and then incubated overnight with anti-digoxigenin antibody coupled to alkaline phosphatase in PBT+20% goat serum. The embryos were then washed extensively before performing an alkaline phosphatase reaction.

### *Xenopus *transgenesis

Human placental alkaline phosphatase (PLAP) cDNA was removed from RISAP vector (C. Cepko, Harvard) by SalI and SpeI and subcloned into XhoI and XbaI site of pCS2^+ ^plasmid to generate CS2-PLAP. CMV promoter was then replaced by the N-β-tubulin (NBT) promoter by SalI and HindIII ligation to generate N-PLAP. Generation of transgenic *X. tropicalis *embryos by restriction enzyme mediated integration (REMI) was carried out according to Kroll and Amaya [22], with the following modifications; 8 × 10^5 ^nuclei of a reaction was diluted in 130 μl of MOH, and injected into eggs using nuclear transplantation needles with an inner diameter between 40–60 μm and a flow rate of 0.2 μl/min; injection buffer was 0.1 × MMR in 6% Ficoll. In each reaction, 100 ng of NotI linearised DNA was used. For generation of the N-PLAP line, REMI transgenesis was performed on, using linearised N-PLAP and γ-crystallin-dsRed, which was modified from? γ-crystallin-GFP [49]. Embryos were grown to the larval stage (St. 47) and assessed for transgenesis by RFP expression in the lens. Those with RFP expression were grown until adulthood. For morpholino experiments, sibling matings between transgenic male and female frogs were performed, and the fertilised embryos were used for injection. We found at least 65% of the F1 embryos expressed PLAP.

### Whole mount alkaline phosphatase reaction

*Xenopus *embryos were fixed with 4% paraformaldehyde in 1 × PBS for 1 hour at room temperature, washed 3 times in 1 × PBS, then endogenous alkaline phosphatase was inactivated by incubation at 65°C for up to 1 hour in 1 × PBS. The embryos were then incubated in AP buffer (100 mM Tris, pH 9.5, 100 mM NaCl, 50 mM MgCl_2_) for 15 minutes followed by AP reaction (NBT/BCIP tablet (Roche)) for 15–30 minutes in the dark. Once staining was detected, the embryos were fixed in MEMFA for 1 hour, and transferred to methanol for storage. For whole mount imaging, embryos were bleached in 1%H_2_O_2_, 5% Formamide and 0.5 × SSC, transferred to methanol, then viewed with Murray's clearing solution (2:1, Benzyl Benzoate:Benzyl alcohol).

### Microdissections

#### *In vivo *cement gland substitution

Capped mRNAs were synthesized using the mMessage mMachine kit (Ambion) for the following cDNAs: mouse BDNF from BDNF-pGem4Z (K. Albers, U Pittsburgh) and GFP from pCXGFP3. Embryos were injected with approximately 250 pg RNAs in the animal pole of both blastomeres at the 2 cells stage according to established protocols. Developmental staging was assessed as described (Nieuwkoop and Faber, 1967). Cement gland deletion and substitution assay were performed as described with modifications [6]. All embryos were transferred to 0.4 × MMR saline solution containing 2% Ficoll for microdissection. Injected embryos at blastula stage (St. 8) and uninjected embryos at neurula stage (St. 18/19) were placed adjacent to one another in small chambers made in agarose-plated petri dishes to keep the embryos in place. Animal caps were dissected from blastula embryos, followed by the removal of the pigmented cement gland from neurula embryos using fine forceps. The caps were then trimmed and fitted onto the cement gland-null neurula embryos. These embryos were kept undisturbed for at least 3 hours following microsurgery to heal, and then transferred to Petri dishes containing 0.1 × MMR until late tailbud stage.

#### *In vivo *cement gland swaps

Antisense morpholino oligonucleotides (20 pg MO BDNFatg or MOC) together with 250 pg mRNA encoding GFP were injected in the animal pole of both blastomeres at the 2 cells stage in *X. laevis *according to established protocols. At the same time, uninjected embryos from the same fertilisation were raised in separate dishes containing 0.1 × MMR. Developmental staging was assessed as described (Nieuwkoop and Faber, 1967). MO injected embryos were confirmed by the detection of GFP fluorescence before the *in vivo *swap experiments. All embryos were transferred to 0.4 × MMR saline solution containing 2% Ficoll for microdissection. MO injected embryos and uninjected embryos at neurula stage (St. 18/19) were placed adjacent to one another in small chambers made in agarose-plated petri dishes to keep the embryos in place. Pigmented cement glands were dissected from both embryos using fine forceps and exchanged with each other. The embryos were then kept undisturbed for at least 3 hours following microsurgery to heal, and then transferred to Petri dishes containing 0.1 × MMR until St.29/30 before fixation and analysis.

## Authors' contributions

JKH and KD contributed equally to this work. JKH conceived the experiments, JKH and KD designed, executed and analysed the experiments, and wrote the paper. SI generated the N-PLAP transgenic *X. tropicalis *line. EA participated in the design and analysis of the study, contributed to the writing of the paper and was the overseer of the project. All authors read and approved the final manuscript.

## Supplementary Material

Additional File 1 A movie showing uninjected *X. tropicalis *embryos (St.32) exhibiting normal mechanosensory response. Click here for file

Additional File 2 A movie showing control morpholino injected *X. tropicalis *embryos (St.32) exhibiting normal mechanosensory response. Click here for file

Additional File 3 A movie showing BDNF morpholino injected *X. tropicalis *embryos (St.32) exhibiting paralysis. Click here for file

Additional File 4 Additional images of *in vivo *cement gland grafts. Click here for file

Additional File 5 A movie showing NT3 morpholino injected *X. tropicalis *embryos (St.32) exhibiting normal mechanosensory response. Click here for file
