# Supplementary figures and images for: BDNF promotes target innervation of Xenopus mandibular trigeminal axons in vivo
Source: BMC Dev Biol. 2007 May 31;7:59. doi: 10.1186/1471-213X-7-59 (PMC1899173; doi:10.1186/1471-213X-7-59)

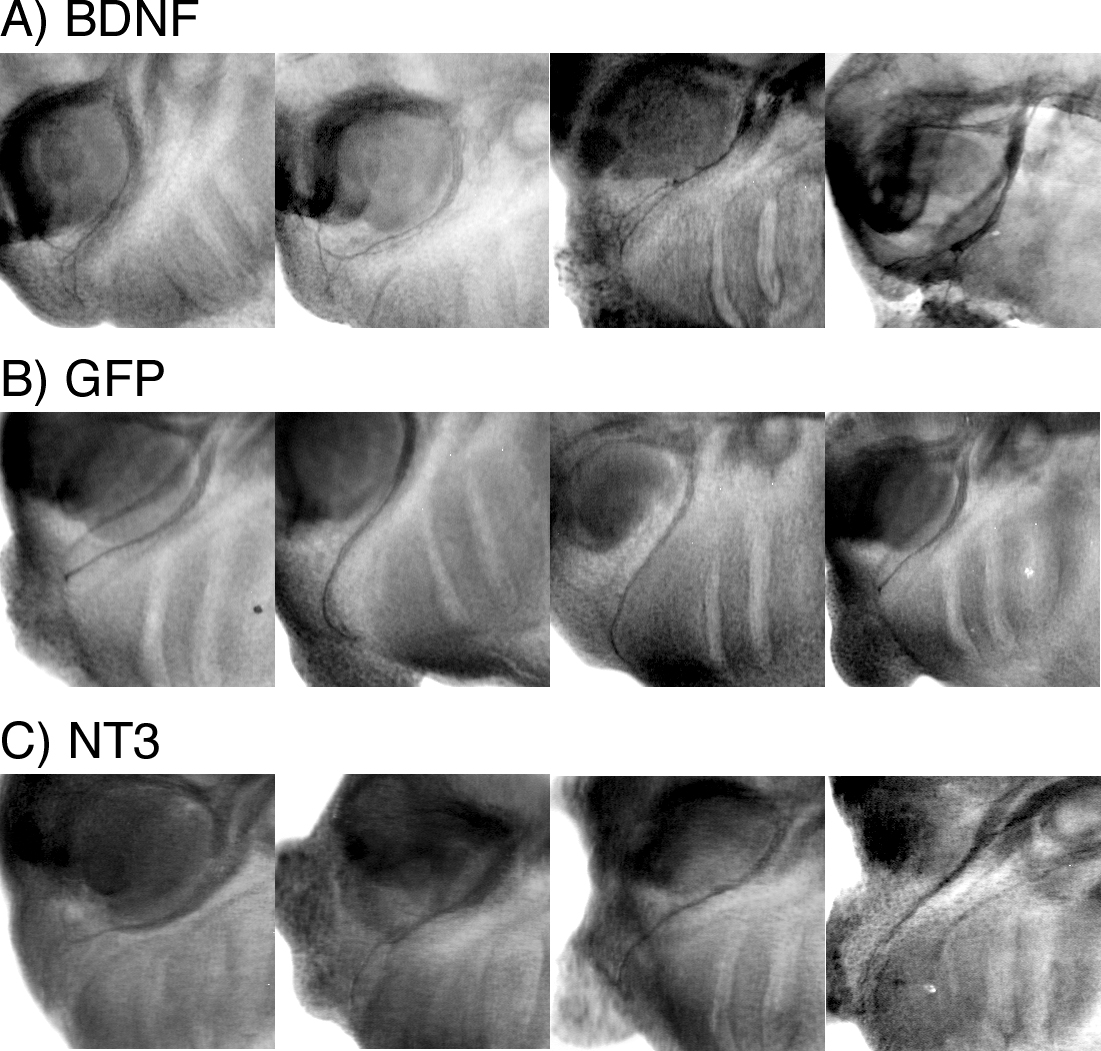

Supplement: Additional File 4 — Additional images of in vivo cement gland grafts. [file 1471-213X-7-59-S4.jpeg]
